# First person – Meg Watt

**DOI:** 10.1242/bio.061812

**Published:** 2024-11-26

## Abstract

First Person is a series of interviews with the first authors of a selection of papers published in Biology Open, helping researchers promote themselves alongside their papers. Meg Watt is co-first author on ‘ Disrupting the interaction between AMBRA1 and DLC1 prevents apoptosis while enhancing autophagy and mitophagy’, published in BiO. Meg conducted the research described in this article while a cell biology research assistant in Kate Hawkins's lab at MSD, London. She is now a PhD student in the lab of Fiona Houston at the Roslin Institute, Easter Bush, Edinburgh, investigating the processes involved in neurodegenerative diseases and potential therapeutic targets and strategies to treat them.


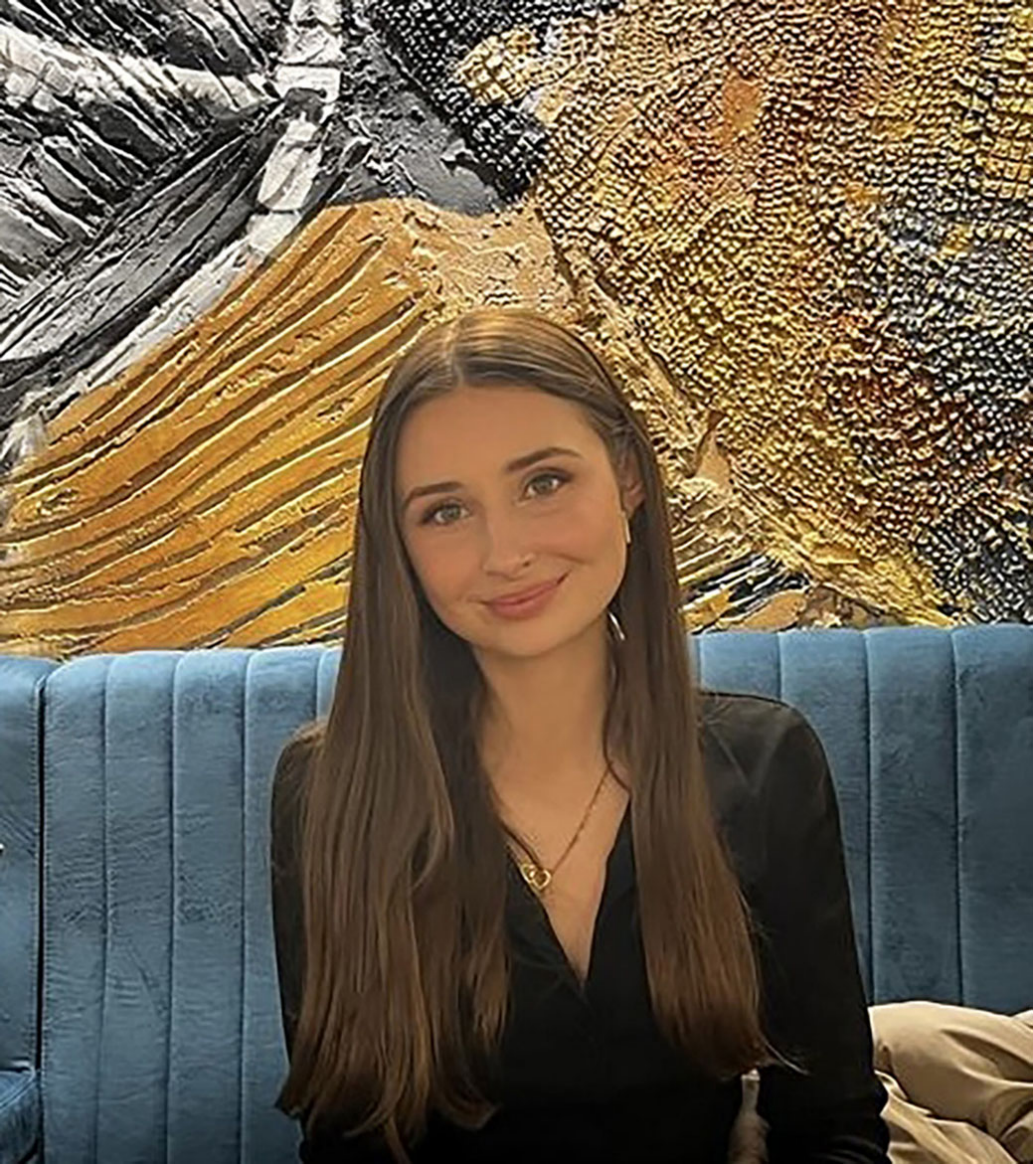


**Meg Watt**

**Describe your scientific journey and your current research focus**

My initial field of interest lay in biochemistry, which led me to study a Bachelor of Science degree in this subject at the University of Bath in the UK. During this degree, I undertook a 12-month placement at the biopharmaceutical company, MSD. My research project was based on using a neuroblastoma cell line to investigate the overactivation of the protein AMBRA1, to initiate a shift away from apoptosis and towards autophagy/mitophagy, a potential strategy for treating neurodegenerative diseases such as Alzheimer's and Parkinson's diseases. This placement further solidified my passion for research, and how I thoroughly enjoyed the investigation into unanswered questions. I made the decision to go straight into studying my current PhD at the Roslin Institute in Edinburgh, investigating neuropathological processes related to ageing in both cats and sheep, along with potential predisposing genetic factors.

**Who or what inspired you to become a scientist?**

At the age of 11, when asked to pick an inspirational person for my summer project, I chose Sally Ride. She was a physicist and in the first class of NASA astronauts to include women, who had a passion for encouraging children, especially girls, into the world of science. The love of science must have already been inspiring me at a young age. My interest in neurological research stemmed from my sister's experience of managing a chronic condition, with little success from medication. The impact this had on her life and of those around her, inspired me to want to make a difference and my passion for the topic of medical neuroscience was ignited.

My interest in neurological research stemmed from my sister's experience of managing a chronic condition, with little success from medication

**How would you explain the main finding of your paper?**

AMBRA1 is a protein that helps with removal of unwanted and damaged cell components and the controlling of cell death. When these processes go wrong, it can lead to neurodegenerative diseases. In this paper we have created mutant versions of AMBRA1 to study how to activate it for potential treatments. A mutant of AMBRA1, which cannot interact with another protein (DLC1) was the most promising. The mutant reduced cell death, increased processes involved in removal of unwanted components and promoted growth in brain cells. Future treatments could focus on blocking AMBRA1's interaction with DLC1 to treat neurodegenerative diseases.

**Figure:**
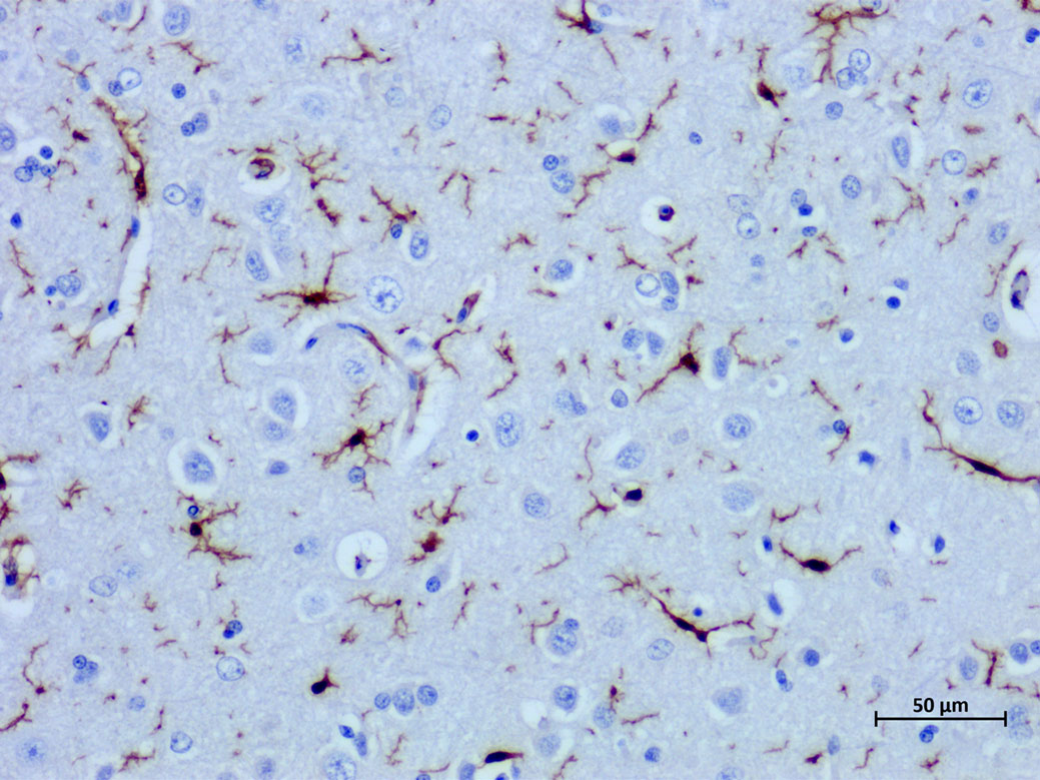
Microglia in the brain of an elderly cat.

**What are the potential implications of this finding for your field of research?**

Neurodegenerative diseases affect millions of people worldwide. Processes that are influenced by AMBRA1 have been implicated in neurodevelopment and neurogenesis. The findings from this paper suggest a potential therapeutic strategy using AMBRA1 to treat neurodegenerative diseases such as Alzheimer's disease and Parkinson's disease.

findings from this paper suggest a potential therapeutic strategy using AMBRA1 to treat neurodegenerative diseases such as Alzheimer's disease and Parkinson's disease

**Which part of this research project was the most rewarding?**

I found the whole experience of working on this research project to be rewarding, from finding novel scientific discoveries, to having the opportunity to hone my skills in an industrial setting. Working alongside Kate Hawkins and tapping into her experience in this field was invaluable. The most rewarding part of the project was presenting my completed results to the senior scientists in the knowledge that I had made a valuable contribution to this field of research.

**What do you enjoy most about being an early-career researcher?**

I enjoy the excitement of daily challenges, and that there is the constant opportunity to learn a new invaluable skill. I find it rewarding that I am exposed to many different research topics and meet people and fellow scientists who have a real passion for such a large range of different research areas.

**What piece of advice would you give to the next generation of researchers?**

Always believe that you do have the potential to be an excellent researcher. Research can seem daunting, especially when you have limited experience. However, the world of research is filled with academics and scientists who want you to succeed, and through your effort, hard work and interactions with your colleagues you will grow to be an amazing researcher, the more you challenge yourself.
